# QTLs and candidate genes analyses for fruit size under domestication and differentiation in melon (*Cucumis melo* L.) based on high resolution maps

**DOI:** 10.1186/s12870-021-02904-y

**Published:** 2021-03-03

**Authors:** Qun Lian, Qiushi Fu, Yongyang Xu, Zhicheng Hu, Jing Zheng, Aiai Zhang, Yuhua He, Changsheng Wang, Chuanqiang Xu, Benxue Chen, Jordi Garcia-Mas, Guangwei Zhao, Huaisong Wang

**Affiliations:** 1 Key Laboratory of Biology and Genetic Improvement of Horticultural Crops of the Ministry of Agriculture and Rural Affairs, Institute of Vegetables and Flowers, Chinese Academy of Agricultural Sciences, 100081 Beijing, China; 2 Shenzhen Branch, Guangdong Laboratory for Lingnan Modern Agriculture, Genome Analysis Laboratory of the Ministry of Agriculture, Agricultural Genomics Institute at Shenzhen, Chinese Academy of Agricultural Sciences, Shenzhen, 518000 China; 3 Henan Key Laboratory of Fruit and Cucurbit Biology, Zhengzhou Fruit Research Institute, Chinese Academy of Agricultural Sciences, Zhengzhou, 450009 China; 4 National Center for Gene Research, CAS Center for Excellence in Molecular Plant Sciences, Shanghai, 200000 China; 5 Shenyang Agricultural University, College of Horticulture, Shenyang, 110866 China; 6 Design Gollege, Zhoukou Normal University, Zhoukou, 466000 China; 7 Centre for Research in Agricultural Genomics CSIC-IRTA-UAB-UB, Barcelona, Spain; 8 Institut de Recerca i Tecnologia Agroalimentàries (IRTA), Barcelona, Spain

**Keywords:** Melon, Genetic map, Fruit size, QTL analysis

## Abstract

**Background:**

Melon is a very important horticultural crop produced worldwide with high phenotypic diversity. Fruit size is among the most important domestication and differentiation traits in melon. The molecular mechanisms of fruit size in melon are largely unknown.

**Results:**

Two high-density genetic maps were constructed by whole-genome resequencing with two F_2_ segregating populations (WAP and MAP) derived from two crosses (cultivated *agrestis* × wild *agrestis* and cultivated *melo* × cultivated *agrestis*). We obtained 1,871,671 and 1,976,589 high quality SNPs that show differences between parents in WAP and MAP. A total of 5138 and 5839 recombination events generated 954 bins in WAP and 1027 bins in MAP with the average size of 321.3 Kb and 301.4 Kb respectively. All bins were mapped onto 12 linkage groups in WAP and MAP. The total lengths of two linkage maps were 904.4 cM (WAP) and 874.5 cM (MAP), covering 86.6% and 87.4% of the melon genome. Two loci for fruit size were identified on chromosome 11 in WAP and chromosome 5 in MAP, respectively. An auxin response factor and a YABBY transcription factor were inferred to be the candidate genes for both loci.

**Conclusion:**

The high-resolution genetic maps and QTLs analyses for fruit size described here will provide a better understanding the genetic basis of domestication and differentiation, and provide a valuable tool for map-based cloning and molecular marker assisted breeding.

**Supplementary Information:**

The online version contains supplementary material available at 10.1186/s12870-021-02904-y.

## Background

Melon (*Cucumis melo* L., 2n = 24) is a very important economic crop with a diverse phenotypic variation in fruit, and is cultivated globally with more than 32 million tons’ yield produced in 2017 (FAOSTAT; http://faostat.fao.org). The market value of melon is influenced by fruit quality in terms of fruit size, fruit shape, flesh color, skin color and flavor, which is mainly determined by sugar content, acidity and the aroma profile [1–3]. Based on ovary pubescence, melon was classified into two subspecies, *Cucumis melo ssp. melo* and *Cucumis melo ssp. agrestis*, and then further divided into 16 horticultural groups according to morphological variations of fruit [1]. *C. melo* ssp. *melo* is cultivated worldwide, whereas *C. melo* ssp. *agrestis* is concentrated in East Asia.

A key step to detect QTLs and perform gene mapping is the construction of a reliable genetic map. In the past, several genetic linkage maps from different populations were constructed using a very limited number of markers as simple sequence repeats (SSRs), amplified fragment length polymorphisms (AFLPs), and random amplified polymorphic DNA (RAPD) [4–8]. These maps were mainly used to detect QTLs for disease-resistance and agronomic traits [9–12]. Melon genome was released in 2012 [13] and significantly improved based on a high-resolution genetic map, which employed 580 single nucleotide polymorphisms (SNPs) anchoring 354.8 Mb sequences [14]. This laid a foundation of high-resolution maps in melon. To integrate information from previous research into the melon draft genome, 836 genetic markers including SSRs and SNPs of the consensus map were mapped onto the improved melon genome [15]. Notably, the rapid advance in next-generation sequencing made it possible to use SNP markers and more accurate genotyping to construct ultra-high-density genetic maps. The recent findings of independent domestication events in melon suggested that SNP discovery in diverse melon botanical groups will advance our understanding of the genetic mechanisms of diversification and domestication as shown in several studies [14–17]. There were genetic maps constructed with SNP markers by genotyping-by-sequencing (GBS) and RNA-Seq to identify QTLs controlling fruit quality [18, 19]. However, the high-density genetic map based on whole-genome resequencing suitable for QTL analysis of domestication traits is unavailable to date. Fruit size is among the most important domestication and differentiation traits in melon. Only few known genes associated with fruit size have been reported in melon [16] although some genes have been identified for disease resistance [20, 21], fruit monoecy [22, 23], flesh color and peel color [24, 25].

Whole-genome resequencing (WGR) is a very useful approach for genetic map construction and fine-mapping of genes. According to our previously research, the two subspecies (*agrestis* and *melo*) were domesticated independently and have a strong population differentiation [16]. So, we constructed two F_2_ mapping populations derived from the crosses ‘JL475’ × ‘YS474’ (cultivated *agrestis* × wild *agrestis*, WAP for domestication analysis) and ‘HG118’ × ‘SD119’ (cultivated *melo* × cultivated *agrestis*, MAP for differentiation analysis). The aim of this study was to construct high-density genetic maps using WGR and explore QTLs in the process of melon domestication and differentiation. These genetic maps will assist future breeding programs by facilitating the design of marker-assisted selection of melon.

## Results

### Analysis of resequencing data and variation calling

We used two F_2_ segregating populations to explore the inherent biological mechanisms. One population was generated from a cross ‘JL475’ (*C. melo* ssp. *agrestis* var. *chinensis*) and a wild *agrestis* accession ‘YS474’ (*C. melo* ssp. *agresti*s var. *agrestis*) (WAP), while the other population was generated from a cross between inbred lines ‘HG118’ (*C. melo* ssp. *melo* var*. chandalak*) and ‘SD119’ (*C. melo* ssp. *agrestis* var. *conomon*) (MAP) (Fig. S1). Both the parents and their progenies were sequenced with an average depth of 29.5× and 7.0× (Table S1), respectively. A total of 597 Gb of clean sequencing data was generated for analysis.

After mapping the short reads against the melon genome, we obtained a comprehensive variation set of the whole genome and observed various distribution of variations among the four parental lines on the genome (Fig. S2). The different genomic landscape of variations may be due to the difference in genomes between the sequenced germplasm and reference genome. To explore the loci among the progeny, we only kept the SNPs that were bi-allelic, homozygous and polymorphic between parents. In the end, 1,871,671 and 1,976,589 SNPs were selected for further analysis in WAP and MAP populations, respectively.

### Construction of high-resolution genetic maps

**Fig. 1:**
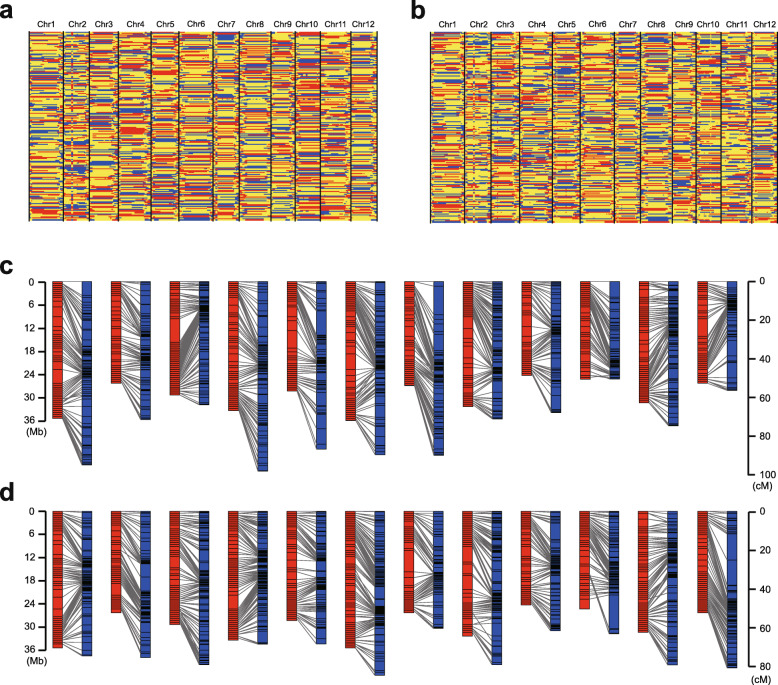
High density genetic maps of WAP and MAP. **a** Recombination map of the cross ‘JL475’ (*C. melo* ssp. *agrestis* var. *chinensis*) and a wild *agrestis* accession ‘YS474’ (*C. melo* ssp. *agresti*s var. *agrestis*) (WAP), which contains 200 individuals. Blue, female (‘JL475’) allele; gray, unknown; yellow, heterozygous; red, male (‘YS474’) allele. **b** Recombination map of the cross between inbred lines ‘HG118’ (*C. melo* ssp. *melo* var*. chandalak*) and ‘SD119’ (*C. melo* ssp. *agrestis* var. *conomon*) (MAP), which contains 200 individuals. Blue, female (‘HG118’) allele; gray, unknown;yellow, heterozygous; red, male (‘SD119’) allele. **c** Integration of physical (left) and genetic (right) maps in WAP. Black short lines represent the genetic and physical positions of the bin markers. **d** Integration of physical (left) and genetic (right) maps in MAP. Black short lines represent the genetic and physical positions of the bin markers

We imported the identified SNPs into a hidden Markov model (HMM) to search recombination events. As a result, 5138 recombination events with an average of 25.7 per individual were observed in WAP (Fig. 1a), and a slightly higher total (5839) and average number (29.2) were observed in MAP (Fig. 1b). We obtained 954 unique bins in WAP (Fig. S3) and 1027 unique bins in MAP (Fig. S4), respectively. All the unique bins were anchored on 12 linkage groups corresponding to 12 chromosomes. The size of bins ranged from 50.2 Kb to 6.3 Mb with an average of 321.3 Kb and covered 86.6% (306.5 Mb) of the anchored melon genome in WAP. Correspondingly, the bins’ size ranged from 30.0 Kb to 5.7 Mb with an average of 301.4 Kb and covered 87.4% (309.6 Mb) of the anchored melon genome in MAP. The genetic distance covered by the unique bins is 904.4 cM in WAP and 874.5 cM in MAP (Table 1). Furthermore, the bin map was integrated with the physical map based on the reference genome, and a high consistency between the genetic and physical positions was observed in both WAP and MAP (Figs. 1c and d), indicating high accuracy of the constructed maps.

**Table 1:** Construction of high-dense genetic maps of WAP (‘JL475’ × ‘YS474’) and MAP (‘HG118’ × ‘SD119’)

Chr	Number of bins		Genetic length (cM)	
	WAP	MAP	WAP	MAP
Chr1	85	86	93.86	74.92
Chr2	74	70	70.57	73.14
Chr3	90	98	63.03	78.66
Chr4	94	109	97.01	68.04
Chr5	64	79	85.78	67.94
Chr6	90	108	88.61	84.08
Chr7	79	64	88.95	66.44
Chr8	91	92	70.30	78.61
Chr9	65	74	67.02	61.25
Chr10	62	73	49.89	62.42
Chr11	99	96	73.76	78.73
Chr12	61	78	55.65	80.26
Total	954	1027	904.43	874.49

### Identification of segregation distortion regions

**Fig. 2:**
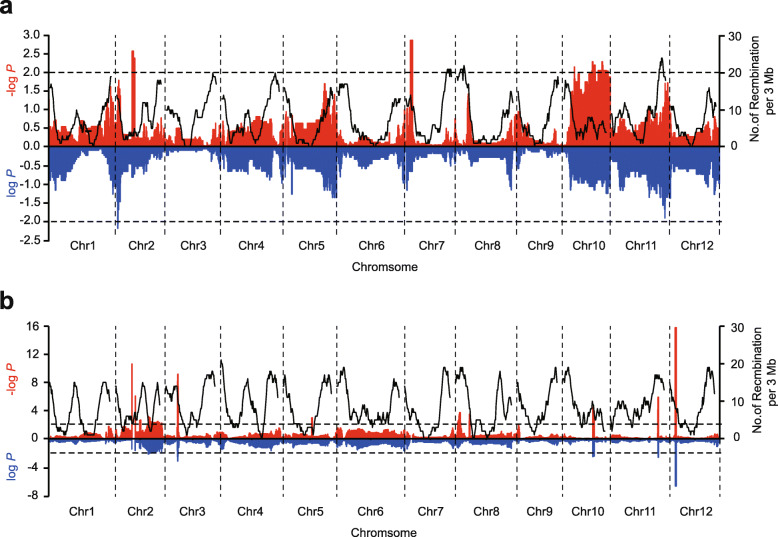
Genome-wide segregation distortions. The segregation distortion regions of bins at the zygotic and gametic stages in the F_2_ populations of WAP (‘JL475’ × ‘YS474’) (**a**) and MAP (‘HG118’ × ‘SD119’) (**b**). The left y-axis represents the –log(*P*) and log(*P*) for the χ^2^ value of each bin at the zygotic (red) and gametic (blue) stages, respectively. The black curves indicate the number of recombination events per 3 Mb. The dash lines show the *P* = 0.01 in the Chi-square test

Segregation distortion regions (SDRs) are frequently found in genetic maps obtained in plants such as potato, cotton and cucumber [26–28]. We also investigated the SDRs of WAP and MAP, both in gametic and zygotic stage (Figs. 2a and b), and observed 7 SDRs with a length of 8.8 Mb in WAP, and 16 SDRs with a length of 10.0 Mb in MAP, respectively. Interestingly, all the SDRs in WAP tend to be present in the male genotype (Table S2), but a different tendency was observed in various SDRs in MAP (Table S3). We further performed gene ontology (GO) analysis to study the functional categories of genes in the SDRs. Genes in SDRs of WAP were enriched in UDP-3-O-[3-hydroxymyristoyl] N-acetylglucosamine deacetylase activity, response to biotic and abiotic stimulus (Table S4), which are associated to plant resistance. However, in MAP, genes located in SDRs were enriched in ‘apoplast’, ‘cell wall’, ‘xyloglucosyl transferase activity’ and ‘cellular glucan metabolic process’ (Table S5), which may be involved in the differentiation of the agronomic traits between *melo* and *agrestis*.

### Validation of the high-resolution mapping with known traits

**Fig. 3:**
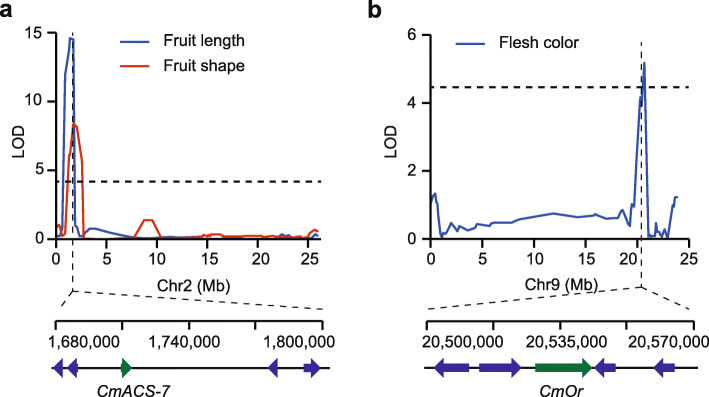
Validation of QTL mapping with known genes (*CmACS-7* and *CmOr*) in WAP (‘JL475’ × ‘YS474’). **a** A multiple-effect QTL was identified for fruit length and fruit shape. *CmACS-7* gene was harbored in the overlapping region (shown with a green arrow). **b** A candidate QTL was identified for flesh color in chromosome 9. The position of the *CmOr* gene was shown with a green arrow

We identified two overlapping QTLs on chromosome 2 in WAP for fruit length and fruit shape with a phenotypic variance explaining of 23.5% (LOD = 14.6) and 13.8% (LOD = 8.4) (Fig. 3a), respectively. Interestingly, we observed that *CmACS7*, encoding an ACC synthase and controlling the monoecy to andromonoecy by an allelic variant [22], was harbored in the overlapping region. Monforte et al. [29] reported that *CmACS7* had pleiotropic effects on fruit length and size, suggesting *CmACS7* might be the logical candidate for the two QTLs. *CmOr* is responsible for the non-orange and orange flesh by inducing β-carotene accumulation in melon [24, 29]. We also identified *CmOr* by QTL-mapping for flesh color with a phenotypic variance explaining of 8.6% (LOD = 5.2) on chromosome 9 in WAP (Fig. 3b). In summary, the above QTLs were mapped precisely or adjacent to known causative genes influencing fruit size and flesh color, demonstrating the reliability of our genetic maps.

### Identification of candidate genes for fruit size

**Fig. 4:**
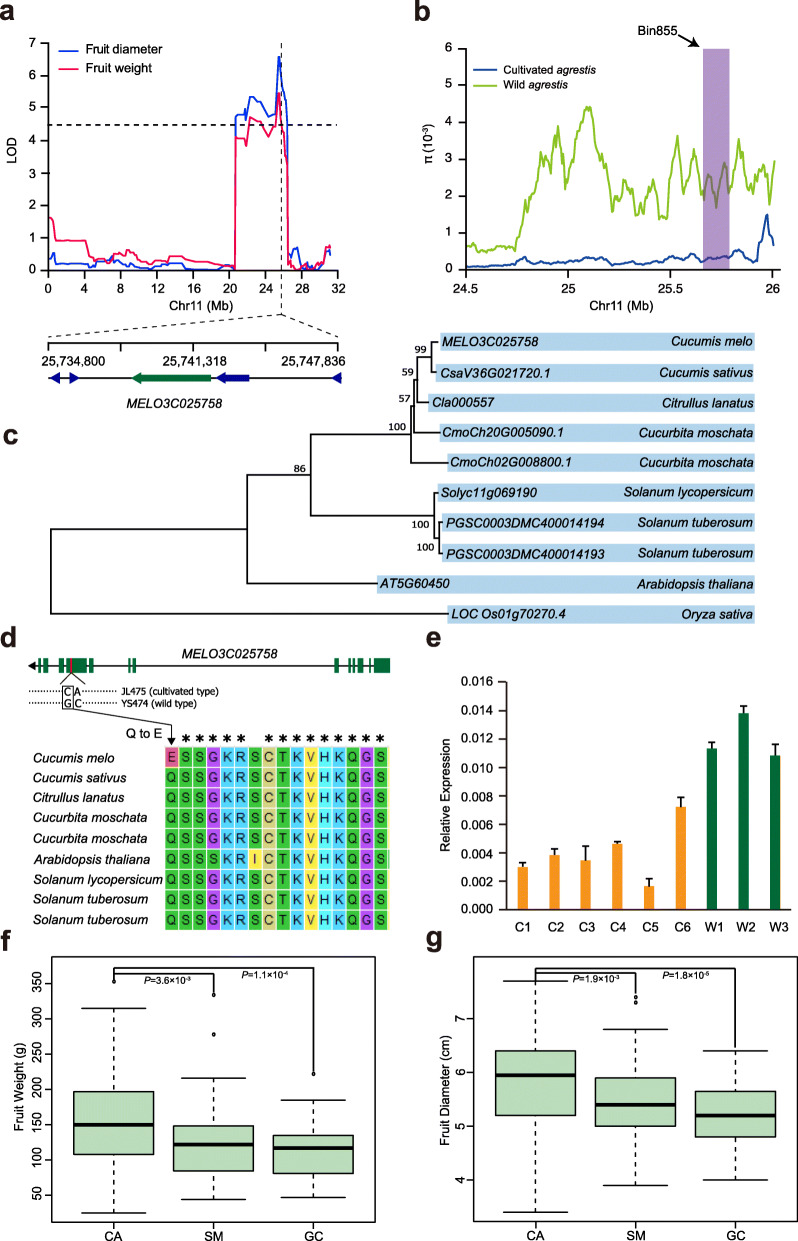
The identification and analysis of *MELO3C025758*. **a** The multiple-effect QTL for fruit weight and fruit diameter in WAP (‘JL475’ × ‘YS474’). **b** Distribution of nucleotide diversity (π) of wild and cultivated agrestis around the bin855. **c** Phylogenetic tree of *MELO3C025758* and its orthologues in rice (*Oryza sativa*), *Arabidopsis* (*Arabidopsis thaliana*), tomato (*Solanum lycopersicum*), potato (*Solanu tuberosum*), pumpkin (*Cucurbita moschata*), watermelon (*Citrullus lanatus*) and cucumber (*Cucumis sativus*). The bootstrap value (100 replications) was listed on the main clades. **d** Gene structure of *MELO3C025758*, line means intron, rectangle means exon and the short red line indicates the position of two adjoint SNPs. Genotyping of the non-synonymous substitution of *MELO3C025758* in six dicots. The genes listed out are all the best hit genes of *MELO3C025758* (*Cucumis sativus: CsaV3_6G021720, Citrullus lanatus: Cla000557, Cucurbita moschata: CmoCh20G005090, Cucurbita moschata: CmoCh02G008800, Arabidopsis thaliana: AT5G60450, Solanum lycopersicum: Solyc11g069190, Solanum tuberosum: PGSC0003DMC400014194, Solanum tuberosum: PGSC0003DMC400014193*). **e** qRT-PCR analysis of *MELO3C025758* in flesh tissue (15 days after pollination), C1-C6 and W1-W3 represent the cultivated and wild accessions, respectively. The distribution of fruit weight (**f**) and fruit diameter (**g**) with three different genotypes that composed by two adjoint SNPs (CA, GC and SM, in which SM means both two SNPs are heterozygous) in WAP, each box represents the mean and interquartile range. *P*-value was marked (T-test, two tail)

We analyzed some important traits (fruit weight, fruit diameter and fruit length) related to fruit size by using the high-resolution and reliable genetic maps. The frequency distributions of fruit weight, fruit diameter and fruit length of the two F_2_ populations obeyed normal distributions (Fig. S5), which indicated they were quantitative traits controlled by multiple nuclear genes. We observed a 130.8 Kb overlapping region on chromosome 11 between two QTLs for fruit diameter (LOD = 6.6) and fruit weight (LOD = 5.5) in WAP, which explained 8.97% and 8.01% of phenotypic variance, respectively (Fig. 4a). Furthermore, the nucleotide diversity of this interval was significantly reduced in the cultivated *agrestis* group compared with wild *agrestis*, suggesting that it may have been selected during melon domestication (Fig. 4b). *MELO3C025758*, encoding an auxin response factor (ARF), was detected in the overlapping region. We also verified this candidate gene using a new method called GradedPool-Seq mapping (GPS-mapping) [30]. This method is modified from bulked-segregant analysis (BSA), and can be used in QTL mapping efficiently. In this method, the F_2_ progenies are classified into several graded bulks according to their phenotypic data, and then, the individuals from each bulk are mixed to provide sufficient genomic coverage for sequencing. Using Ridit analysis, the *p* value between each variation and graded pool is calculated. After filtering and noise removal, the QTLs related to the target phenotypes are mapped. As a result, we also identified *MELO3C025758* in the QTLs of both fruit weight (Fig. S6a) and fruit diameter (Fig. S6b) with GPS-mapping analysis.

To further exploit the function of this gene, we searched the orthologs of *MELO3C025758* in other species as orthologous genes are generally assumed to retain equivalent functions in different organisms [31]. As a consequent, we selected the best hits for ortholog genes from seven plant species to construct a phylogenetic tree (Fig. 4c). We found that the orthologs *AT5G60450* in *Arabidopsis*, *Solyc11g069190* in tomato and *Cla000557* in watermelon were involved in the regulation of fruit development [32–34].

With the resequencing data, we also found two adjoint SNPs (Chr11:25,735,961 and Chr11:25,735,962) that showed polymorphism between ‘JL475’ (cultivated *agrestis*) and ‘YS474’ (wild *agrestis*) (Fig. 4d) and totally linked in F_2_ progenies, that located in the tenth exon of *MELO3C025758*. The former SNP (Chr11:25,735,961) leads to a non-synonymous mutation from Gln (Q) to Glu (E), which located in a conserved domain in dicotyledon that we selected to construct the phylogenetic tree. So, we think this mutation in a conserved domain may affect the function of *MELO3C025758*; nevertheless, we recognize that further experiments are required to verify such assumption. Besides the non-synonymous mutation in a conserved domain, we hypothesize that these two adjoint SNPs may affect the expression of *MELO3C025758.* To verify this hypothesis, we analyzed the expression pattern of this gene in flesh tissue (15 days after pollination) in 9 diverse melon accessions including 6 cultivated *agrestis* (Chinese landraces from the group *C. melo* ssp. *agrestis* var. *chinensis*) and 3 wild *agrestis* accessions (PI 532829, PI 406737, PI 536473). As a result, *MELO3C025758* exhibited a higher expression in wild *agrestis* than that in cultivated *agrestis* (Fig. 4e), and the expression levels had a significant negative correlation with fruit weight (*R*^*2*^ = 0.92) (Fig. S7a) and fruit diameter (*R*^*2*^ = 0.71) (Fig. S7b). Furthermore, the individuals in F_2_ population with cultivated type in the adjoint SNPs showed significantly higher phenotype values than those individuals with wild type both in fruit weight and fruit diameter (Figs. 4f and g). To summarize, *MELO3C025758* may be a candidate gene that play a key role in melon fruit size and further investigation is required to understand its function.

**Fig. 5:**
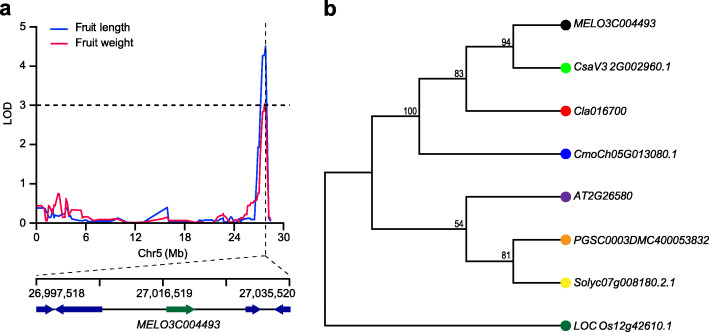
The identification and analysis of *MELO3C004493*. **a** The multiple-effect QTL for fruit weight and fruit length in MAP (‘HG118’ × ‘SD119’). **b** Phylogenetic tree of *MELO3C004493* (black dot) and its orthologues in rice (dark green dot), Arabidopsis (purple dot), tomato (yellow dot), potato (orange dot), pumpkin (blue dot), watermelon (red dot) and cucumber (green dot)

In addition, we identified a QTL for fruit weight (LOD = 3.06) and fruit length (LOD = 4.30), covering a region of 115.9 Kb on chromosome 5 in MAP, which explained 6.01% and 8.01% of phenotypic variance, respectively. In this region, *MELO3C004493* encoded a YABBY transcription factor (Fig. 5a). Its orthologues *AT2G26580* in *Arabidopsis*, *Solyc07g008180.2.1* in tomato, and *LOC Os12g42610.1* (*OsYABBY6*) in rice were previously reported to be involved in the regulation of plant growth [35–37] (Fig. 5b). Additionally, YABBY transcription factors have been reported to be associated with tomato fruit size [36, 38]. Therefore, *MELO3C004493* might be a candidate gene for the fruit size in chromosome 5.

## Discussion

Genetic linkage maps are important tools for studying the genetic regularity of agronomic traits and the genome structure. The first molecular marker linkage map of melon was reported in 1996 mainly using of random amplified polymorphic DNA (RAPD) and restriction fragment length polymorphism (RFLP) markers, but the markers did not cover the 12 melon chromosomes [39]. In the past, a number of molecular genetic maps were constructed [11, 15, 17, 19, 29, 40, 41]. The rapid development of molecular biology techniques and next-generation sequencing have accelerated the construction of genetic maps in melon. WGR is a genotyping method based on sequencing that was used to detect recombination breakpoints and construct genetic maps, which is more accurate compared with marker-based genotyping methods [42–44]. In this study, we constructed two high-density genetic maps based on WGR with millions of SNPs that show differences between parents of ‘JL475’ × ‘YS474’ (WAP) and ‘HG118’ × ‘SD119’ (MAP). A high consistency between the genetic and physical positions was observed in both WAP and MAP. Furthermore, two reported genes *CmACS7* for monoecy and *CmOr* for flesh color were located in or adjacent to the regions of QTLs we mapped [22, 24, 29], suggesting that the high-density markers in the genetic maps using WGR could be efficient in QTL mapping.

Segregation distortion violates the classic separation of Mendelian law, showing a deviation between genotypic frequencies and their expected values [45]. It is a quite common phenomenon in nature and was identified as a powerful evolutional force [46]. Some factors including mapping population, relationships between the parents, and marker types could positively influence the segregation distortion [47]. Segregation distorted loci tend to be clustered and form SDRs. Next-generation sequencing provided a huge number of SNPs to construct ultra-high-density genetic maps, which make it possible to scan SDRs with high resolution. In the present study, we found that 7 SDRs contained 14 bins in WAP and chromosome 10 contained the highest proportion of bins with SDRs (1 SDR in chr 2, 1 SDR in chr 7, 5 SDRs in chr 10) (Table S2). Meanwhile, we observed 16 SDRs with 41 bins in MAP, and most of the SDRs existed on chromosome 2 (6 SDRs in chr 2, 1 SDR in chr 3, 1 SDR in chr 5, 3 SDRs in chr 8, 1 SDR in chr 9, 2 SDR in chr 10, 1 SDR in chr 11, 1 SDR in chr 12) (Table S3). Interestingly, all the SDRs in WAP tend to the male genotype, but a different trend was observed in various SDRs in MAP. We further performed gene ontology (GO) analysis to study the functional categories of the genes within the SDRs. Genes in SDRs of WAP were associated with plant resistance to environmental stresses, which may provide clues for further exploring related resistant genes. However, in MAP, genes located in SDRs were involved in plant growth and development, which might contribute to the determination of diverse agronomic traits between *melo* and *agrestis*. Ren et al. [47] also reported that all markers within the SDRs on chromosomes 1, 2, 3, 4, 5, 7, 8 and 9 were related to the cultivated parent and markers within the SDRs on chromosome 10 were tend to the wild parent. The genomic differences between cultivated and wild types may cause the severe segregation distortion. Segregation distortion in plants may be connected with the genetic background of parental lines used for population construction. Some researchers have suggested that segregation distortion is related with genetic hitch-hiking effect, which means that the frequencies of genes closely linked with the selected genes [48, 49]. The evolution of segregation distortion changes the allele frequency and genotype frequency from generation to generation, which would ultimately lead to reproductive isolation and speciation. The genes located in segregation distortion regions can contribute to improve the adaptation to the environment during the evolution [49].

Fruit size is one of the most important domestication and differentiation traits in melon, and seems to be a more complex controlling by polygenes. Pan et al. [50] summarized 105 fruit size (FS), 103 fruit shape index (FSI), and 57 fruit weight (FW) QTLs in melon from 19 previous studies. The QTLs were distributed across all 12 chromosomes (the most in Chr6 and Chr8), which seems consistent with the higher genetic diversity of melon fruits [11, 17, 19, 29, 50]. However, many of these QTLs were detected in a few studies, and the physical intervals for most QTLs are still very large. The populations in previous studies were commonly originated from the crosses between members of cultivated *melo* and *agrestis* [10, 11, 19, 29]. Though, Díaz et al. [17] used a F_2_ segregating population derived from a cross of wild *agrestis* and cultivated *melo* for QTL-mapping of domestication-related traits with 128 SNP-based markers. Given the independent domestication in subspecies *agrestis* and *melo*, it is meaningful to perform QTLs using the population derived from the wild and cultivated in the same subspecies. A higher resolution QTL analysis map is still needed, and the genetic research of domestication and differentiation still needs to reach the gene level.

In our study, we performed QTL analysis for fruit size with two F_2_ populations derived from two crosses (cultivated *agrestis* × wild *agrestis* and cultivated *melo* × cultivated *agrestis*). These two crosses are good choice for studying the genetics of melon domestication and differentiation. We identified a major QTL for fruit size on chromosome 11 in WAP, a gene *MELO3C025758* (encodes an auxin response factor) was located in the overlapping region for fruit weight and fruit diameter (Fig. 4a). Auxin is essential in determining final fruit size through the control of cell division and cell enlargement [32, 51, 52]. The most rapid period of cell enlargement in melon is 10–15 days after pollination. So, we chose 15 days after pollination to perform qRT-PCR of *MELO3C025758* under our cultivated environment. We found that *MELO3C025758* exhibited a higher expression in wild accessions than in cultivated accessions, and a negative correlation with fruit diameter and fruit weight (Fig. S7), suggesting it might have been selected during melon domestication. A number of researches have demonstrated the role of specific auxin response factors in early stages of fruit development [53–55]. We also identified a candidate gene encoding a YABBY transcription factor (*MELO3C004493*) in a 115.9 Kb QTL interval for fruit size on chromosome 5 in the MAP population (Fig. 5a). The YABBY-like transcription factor has been reported to be related to the evolution of tomato fruit size during domestication, suggesting it may play an important role in fruit size of melon [29, 36, 38]. Therefore, the identified gene *MELO3C004493* is a good candidate gene for fruit size in melon. In addition, the same candidate gene was identified with ‘GradedPool-Seq mapping analysis’ [30] in this study, suggesting that ‘GradedPool-Seq mapping analysis’ could be used as an alternative strategy for QTL analysis and gene discovery. Further studies are nevertheless necessary to corroborate our hypotheses on the importance of both genes to melon domestication and differentiation. Taken together, our study presents a reliable genetic map, which will ultimately support future melon breeding projects using marker-assisted selection.

## Conclusions

In this study, we constructed two high-density genetic maps (WAP and MAP) using whole-genome resequencing. 1,871,671 and 1,976,589 high quality SNPs with a total of 5138 and 5839 recombination events were obtained in WAP and MAP respectively. The total lengths of two linkage maps were 904.4 cM (WAP) and 874.5 cM (MAP), covering 86.6 and 87.4% of the melon genome. Two loci for fruit size were identified on chromosome 11 in WAP and chromosome 5 in MAP. *MELO3C025758* and *MELO3C004493* were inferred to be the candidate genes for both loci. The QTLs analyses of fruit size-related traits will provide further insights into the genetic mechanisms of melon.

## Methods

### Plant materials management

The mapping populations were developed from a cross between ‘JL475’ (*C. melo* ssp. *agrestis* var. *chinensis*) and wild melon ‘YS474’ (*C. melo* ssp. *agresti*s var. *agrestis*) (WAP), and a cross between ‘HG118’ (*Cucumis melo ssp. melo* var*. chandalak*) and ‘SD119’ (*C. melo* ssp. *agrestis* var. *conomon*) (MAP). The parental lines, F_1_ and two F_2_ populations were grown in the spring–summer season of 2017 under greenhouse conditions in the Institute of Vegetables and Flowers, Chinese Academy of Agricultural Sciences, Beijing. Plants were grown under natural light conditions. The green-house was maintained at daily temperatures between 17 and 33 °C, the relative humidity of day/night was about 55/85%. Flowers were artificial pollinated and tagged at anthesis to register the total days of fruit ripening. Two fruits were allowed to develop per plant in WAP, and one fruit was allowed to develop per plant in MAP. Two hundred individuals in each segregating F_2_ population were used for mapping.

‘JL475’ is a Chinese cultivated *agestis* melon accession with white peel and light orange flesh. ‘YS474’ is an Indian wild *agestis* genotype with small fruit (about 30 g). ‘HG118’ is a Chinese inbred line from the group *chandalak*, with round fruits, yellow peel, light green flesh and pleasant fruit aroma. ‘SD119’ is a Chinese landrace from the group *conomon*, with elongated fruits, with non-sweet, white peel and fruit flesh. (Fig. S1).

### Phenotypic and genetic analysis

Mature fruits were weighed with a balance (0.1 g) and cut longitudinally in the middle for measuring fruit diameter (mm) and fruit length (mm) with a ruler. Flesh color were evaluated visually following a technical specification for evaluating melon [16]. Frequency distribution analysis and graphic representations were performed using SPSS (24.0) software and R program v3.1.2, respectively.

### Genome sequencing

Genomic DNA was extracted from young leaves based on the CTAB procedure [56]. Whole-genome paired-end sequencing was performed on the Illumina HiSeq X Ten platform, with a library insert size of 250–300 bp and a read length of 150 bp.

### Recombination map construction

After removing the adapter and filter out low quality reads, we mapped the short reads against the reference genome [14] with BWA-aln, using the default parameters [57]. The samtools and bcftools programs were applied to generate the genotype of all the loci on the genome, with the default settings [58].

Firstly, we detected loci showing bi-allelic, homozygous and polymorphic between parents, then, we filtered out the loci with the following criteria: quality ≥20, MQ ≥ 20. Secondly, the genotypes of above-mentioned loci were extracted from F_2_ individuals. To avoid the bias that may be brought by gene conversion or sequencing error, a hidden Markov model was used to impute genotypes of recombinant chromosome fragments for all the F_2_ individuals based on observed genotypes of SNPs as described previously [59]. A region located between two adjacent blocks with different genotypes was defined as a crossover. Finally, the bin map was constructed based on the boundary of all recombinant chromosome fragments and the bins with length below 30 Kb were excluded to avoid false recombination [28].

### Genetic map construction

The software MSTMap was used for constructing a linkage map with the parameters “*p* value = 0.0000001, missing threshold = 0.2, distance function = kosambi, objective function = ML” [60].

### QTL analysis

QTL analysis was performed using R/qtl, a software package for mapping quantitative trait loci [58]. We selected the composite interval mapping with the *cim* function. LOD thresholds determined based on the 1000 permutations (*P* < 0.05) for every trait with the function of permutation in R/qtl. The support interval of a QTL was defined by the region of peak LOD bin and the bilateral bins. The variation explained and the additive effect of each QTL detected from the data set was estimated using the fitqtl function. QTLs with overlapping support intervals for the same trait were considered as a single QTL.

### GradedPool-Seq mapping analysis

According to the recommendation from Wang et al. [30], it is appropriate to select 20–30% total individuals as a bulk. Firstly, we ranked the phenotype values from high to low. Then, we divided all the ranked individuals into four pools with the criteria that about a quarter of total individuals in each pool. According to this criterion, the F_2_ populations were pooled based on the phenotype values from low to high both in WAP (Fruit weight: 25-85 g, 86-126 g, 127-160 g, 162-353 g. Diameter: 3.0–4.9 cm, 5.0–5.5 cm, 5.6–6.0 cm, 6.1–7.8 cm) and MAP (Fruit weight: 300-628 g, 630-777 g, 782-1059 g, 1060-1942 g. Fruit length: 7.9–14.5 cm, 14.6–16.7 cm, 16.8–18.9 cm, 19-28 cm). Next, we merged their sequencing data for every mixed pool, in this way, we aligned the merged reads to reference genome and calculated the depth for each variant. After filtering the variants with low quality and depth using default parameters, we calculated the *p*-values with Ridit analysis for each variant. At last, to reduce the background noise, we set the sliding window size to 400 Kb and the candidate region was set as the peak interval and the 200 Kb region extant from both sides as reported by Wang et al. [30]. All the codes and parameters about GPS-mapping analysis are available in GitHub (https://github.com/sctang1991/ GPS-pipeline).

### Phylogenetic tree construction

We selected the best hit genes amino acid sequence in *Arabidopsis*, rice, potato, tomato, pumpkin, watermelon and cucumber using blast with default settings. The multiple sequence alignment was obtained using clustalW in MEGA 6.0 software [61]. Next, the maximum likelihood algorithm was used to construct the phylogenetic tree with 100 of bootstrap value in MEGA [62].

### Nucleotide diversity analysis

Taking advantage of the whole genome result of nucleotide diversity analysis from Zhao et al. [16], we selected the target region around our candidate genes and graphed it with R script.

### Quantitative real-time PCR analysis (qRT-PCR)

Fruit flesh samples (about 0.2 g) were collected 15 days after pollination, frozen immediately in liquid nitrogen and stored at − 80 °C until use for RNA extraction. Total RNA was extracted from the flesh as described in the TRI reagent protocol (Takara Bio Inc., Japan). For all samples, total RNA (1 μg) was converted to cDNA using PrimeScript™ 1st Strand cDNA Synthesis Kits (Takara) according to the manufacturer’s instructions. Specific primers were designed using Primer Premier 6.0 (http://www.premierbiosoft.com/primerdesign/index.html). Gene-specific primers used for qRT-PCR were shown in Table 2. All reactions were performed with SYBR PrimeScript™ RT-PCR Kits (Takara Bio Inc., Shiga, Japan) according to the manufacturer’s instructions. Quantitative RT-PCR was conducted with a LightCycler® 96 Instrument (Roche, Mannheim, Germany).

**Table 2:** Primers used to amplify the target genes for qRT-PCR analysis

Gene	Forward primer	Reverse primer
*MELO3C025758*	ATGTTCACCTACTCGCCAATAAG	CTCCATTCAACGC CAATTCCT
*Actin*	TTACGGAAACATCGTCCTCAG	GAATAGACCCTCCAATCCAAAC

## Supplementary Information

**Additional file 1: Figure S1**. The fruit images used in the two populations. Mapping populations were developed from a cross between ‘HG118’ (*Cucumis melo ssp. melo* var*. chandalak*) and ‘SD119’ (*C. melo* ssp. *agrestis* var. *conomon*) (MAP), and a cross between ‘JL475’ (*C. melo* ssp. *agrestis* var. *chinensis*) and wild melon ‘YS474’ (*C. melo* ssp. *agresti*s var. *agrestis*) (WAP). The scale bar means 1 cm. **Figure S2**. The genome landscape of variations in the parental lines. **Figure S3**. The distribution of bins in WAP (‘JL475’ × ‘YS474’). **Figure S4**. The distribution of bins in MAP (‘HG118’ × ‘SD119’). **Figure S5**. Frequency distributions of fruit weight, fruit length, and fruit diameter in two F_2_ populations. **Figure S6**. GPS-mapping of *MELO3C025758* in WAP. **Figure S7**. The correlation between gene expression (*MELO3C025758*) and fruit weight **(A)** and fruit diameter **(B)** in 9 diverse melon accessions including 6 cultivated *agrestis* (Chinese landraces from the group *C. melo* ssp. *agrestis* var. *chinensis*) (orange dot) and 3 wild *agrestis* accessions (green dot). **Table S1**. The summary of sequencing statistics. **Table S2**. The SDRs in WAP (‘JL475’ × ‘YS474’). **Table S3**. The SDRs in MAP (‘HG118’ × ‘SD119’). **Table S4**. The result of GO enriches of genes in SDRs in WAP (‘JL475’ × ‘YS474’) **Table S5**. The result of GO enriches of genes in SDRs in MAP (‘HG118’ × ‘SD119’).

## Data Availability

The datasets used and/or analysed during the current study are available from the corresponding author on reasonable request, and all the sequencing data were submitted to NCBI Sequence Read Archive database under accession number PRJNA685284.

## Abbreviations

QTL: Quantitative trait locus
Chr: Chromosome
qRT-PCR: Quantitative real-time PCR
SNP: Single nucleotide polymorphism
Indel: Insertion-deletion
SSR: Simple sequence repeat
RAPD: Random amplified polymorphic DNA
RFLP: Restriction fragment length polymorphism
LG: Linkage group
LOD: Logarithm of odds
cM: centiMorgan
GBS: Genotyping-by-sequencing
WGR: Whole-genome resequencing
SDR: Segregation distortion regions
GO: Gene ontology
