# Supplementary material for: QTLs and candidate genes analyses for fruit size under domestication and differentiation in melon (Cucumis melo L.) based on high resolution maps
Source: BMC Plant Biol. 2021 Mar 3;21:126. doi: 10.1186/s12870-021-02904-y (PMC7931605; doi:10.1186/s12870-021-02904-y)
Supplement: Supplementary file 1 — Additional file 1: Figure S1. The fruit images used in the two populations. Mapping populations were developed from a cross between ‘HG118’ (Cucumis melo ssp. melo var. chandalak) and ‘SD119’ (C. melo ssp. agrestis var. conomon) (MAP), and a cross between ‘JL475’ (C. melo ssp. agrestis var. chinensis) and wild melon ‘YS474’ (C. melo ssp. agrestis var. agrestis) (WAP). The scale bar means 1 cm. Figure S2. The genome landscape of variations in the parental lines. Figure S3. The distribution of bins in WAP (‘JL475’ × ‘YS474’). Figure S4. The distribution of bins in MAP (‘HG118’ × ‘SD119’). Figure S5. Frequency distributions of fruit weight, fruit length, and fruit diameter in two F2 populations. Figure S6. GPS-mapping of MELO3C025758 in WAP. Figure S7. The correlation between gene expression (MELO3C025758) and fruit weight (A) and fruit diameter (B) in 9 diverse melon accessions including 6 cultivated agrestis (Chinese landraces from the group C. melo ssp. agrestis var. chinensis) (orange dot) and 3 wild agrestis accessions (green dot). Table S1. The summary of sequencing statistics. Table S2. The SDRs in WAP (‘JL475’ × ‘YS474’). Table S3. The SDRs in MAP (‘HG118’ × ‘SD119’). Table S4. The result of GO enriches of genes in SDRs in WAP (‘JL475’ × ‘YS474’) Table S5. The result of GO enriches of genes in SDRs in MAP (‘HG118’ × ‘SD119’). [file 12870_2021_2904_MOESM1_ESM.docx]

**QTLs and Candidate Genes Analyses for Fruit Size under Domestication and Differentiation in Melon (*Cucumis melo* L.) Based on High Resolution Maps**

**Qun Lian^1,2+^, Qiushi Fu^1+^, Yongyang Xu^3^, Zhicheng Hu^1^, Jing Zheng^1^, Aiai Zhang^1^, Yuhua He^3^, Changsheng Wang^4^, Chuanqiang Xu^5^, Benxue Chen^6^, Jordi Garcia-Mas^7,8^, Guangwei Zhao^3*^ and Huaisong Wang^1*^**

^1^ Institute of Vegetables and Flowers, Chinese Academy of Agricultural Sciences, Beijing 100081, China.

^2^ Shenzhen Branch, Guangdong Laboratory for Lingnan Modern Agriculture, Genome Analysis Laboratory of the Ministry of Agriculture, Agricultural Genomics Institute at Shenzhen, Chinese Academy of Agricultural Sciences, Shenzhen 518000, China.

^3^ Henan Key Laboratory of Fruit and Cucurbit Biology, Zhengzhou Fruit Research Institute, Chinese Academy of Agricultural Sciences, Zhengzhou 450000, China.

^4^ National Center for Gene Research, CAS Center for Excellence in Molecular Plant Sciences, Shanghai 200000, China.

^5^ Shenyang Agricultural University, College of Horticulture, Shenyang 110866, China.

^6^ Design Gollege, Zhoukou Normal University, Zhoukou 466000, China.

^7^ Centre for Research in Agricultural Genomics CSIC-IRTA-UAB-UB, Barcelona, Spain.

^8^ Institut de Recerca i Tecnologia Agroalimentàries (IRTA), Barcelona, Spain.

^*^ Correspondence:

Huaisong Wang, [wanghuaisong06@sina.com](mailto:wanghuaisong06@sina.com); Tel.: +86-010-82105984;

Guangwei Zhao, Zhaoguangwei@caas.cn; Tel.: +0371-0371-65330931

^+^ These authors contributed equally: Qun Lian, Qiushi Fu.


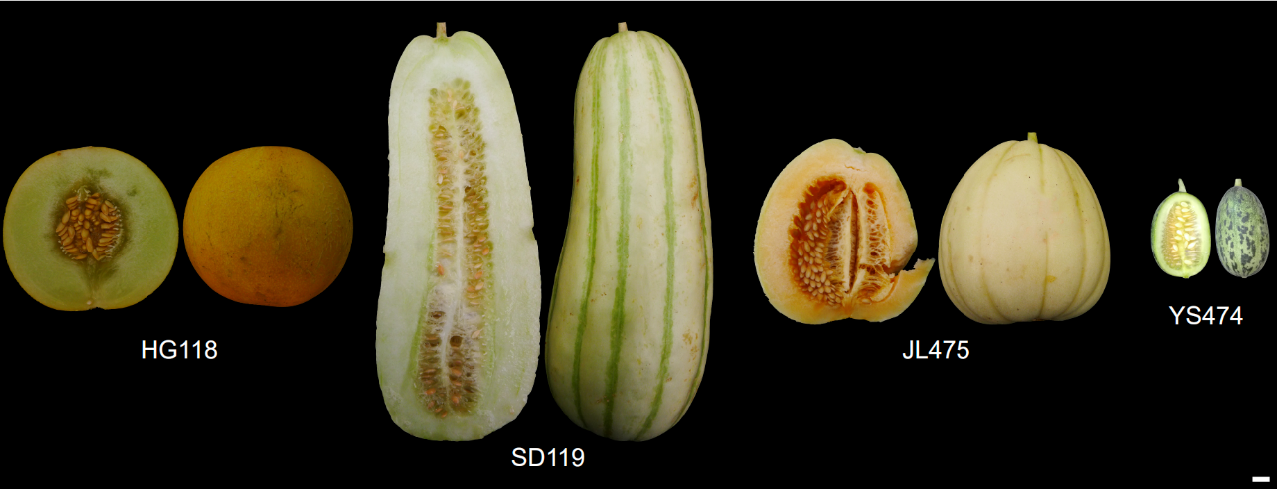


**Fig. S1** The fruit images used in the two populations. Mapping populations were developed from a cross between ‘HG118’ (*Cucumis melo* ssp. *melo* var*. chandalak*) and ‘SD119’ (*C. melo* ssp. *agrestis* var. *conomon*) (MAP), and a cross between ‘JL475’ (*C. melo* ssp. *agrestis* var. *chinensis*) and wild melon ‘YS474’ (*C. melo* ssp. *agresti*s var. *agrestis*) (WAP). The scale bar means 1 cm.


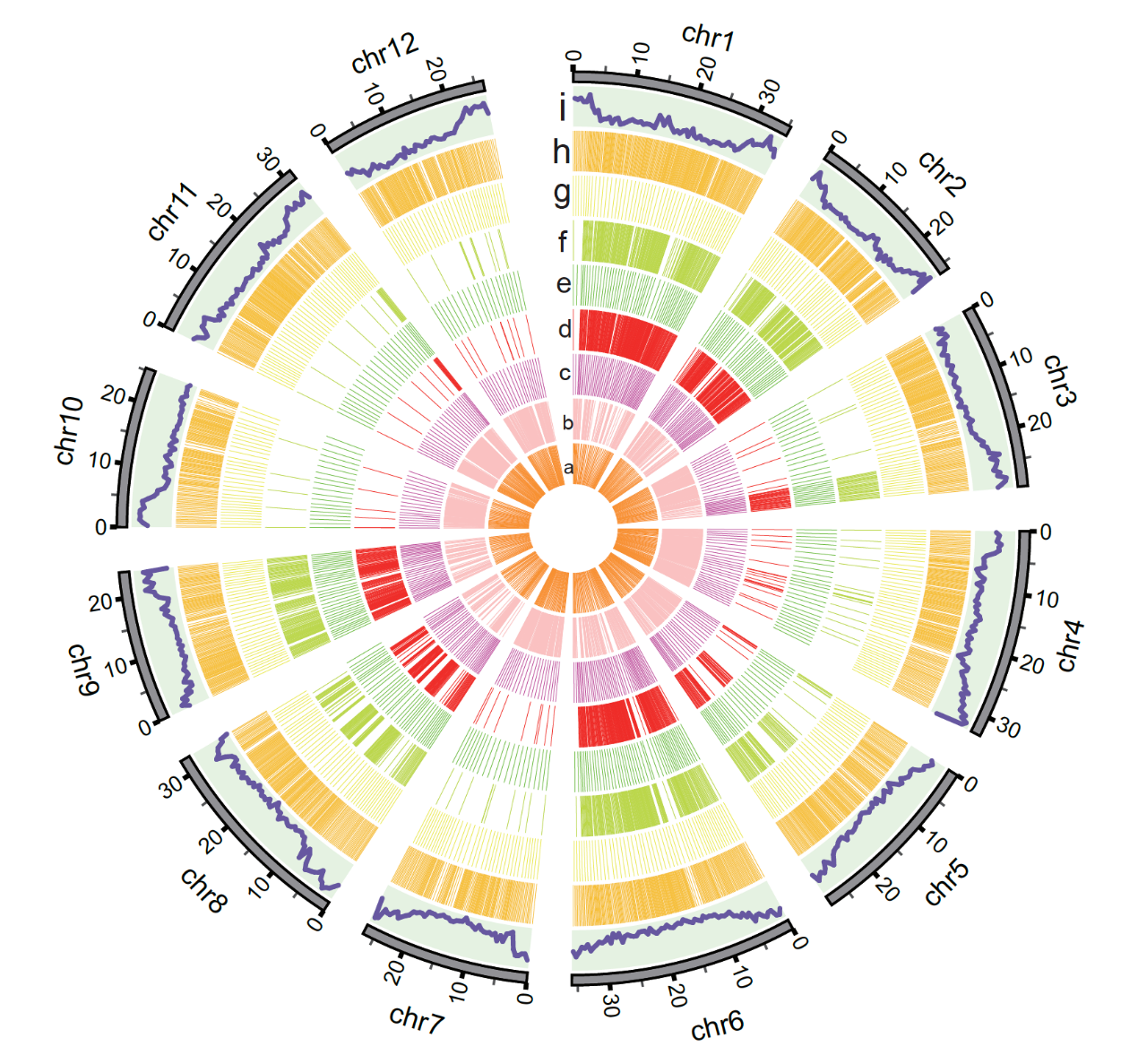


**Fig. S2** The genome landscape of variations in the parental lines.

**a-i:** A short lines present a window including 1000 variations. Indels **(a)** and SNPs **(b)** in female of MAP (‘HG118’ × ‘SD119’), Indels **(c)** and SNPs **(d)** in male of MAP, Indels **(e)** and SNPs **(f)** in female of WAP (‘JL475’ × ‘YS474’), Indels **(g)** and SNPs **(h)** in male of WAP, Gene density **(i)**.


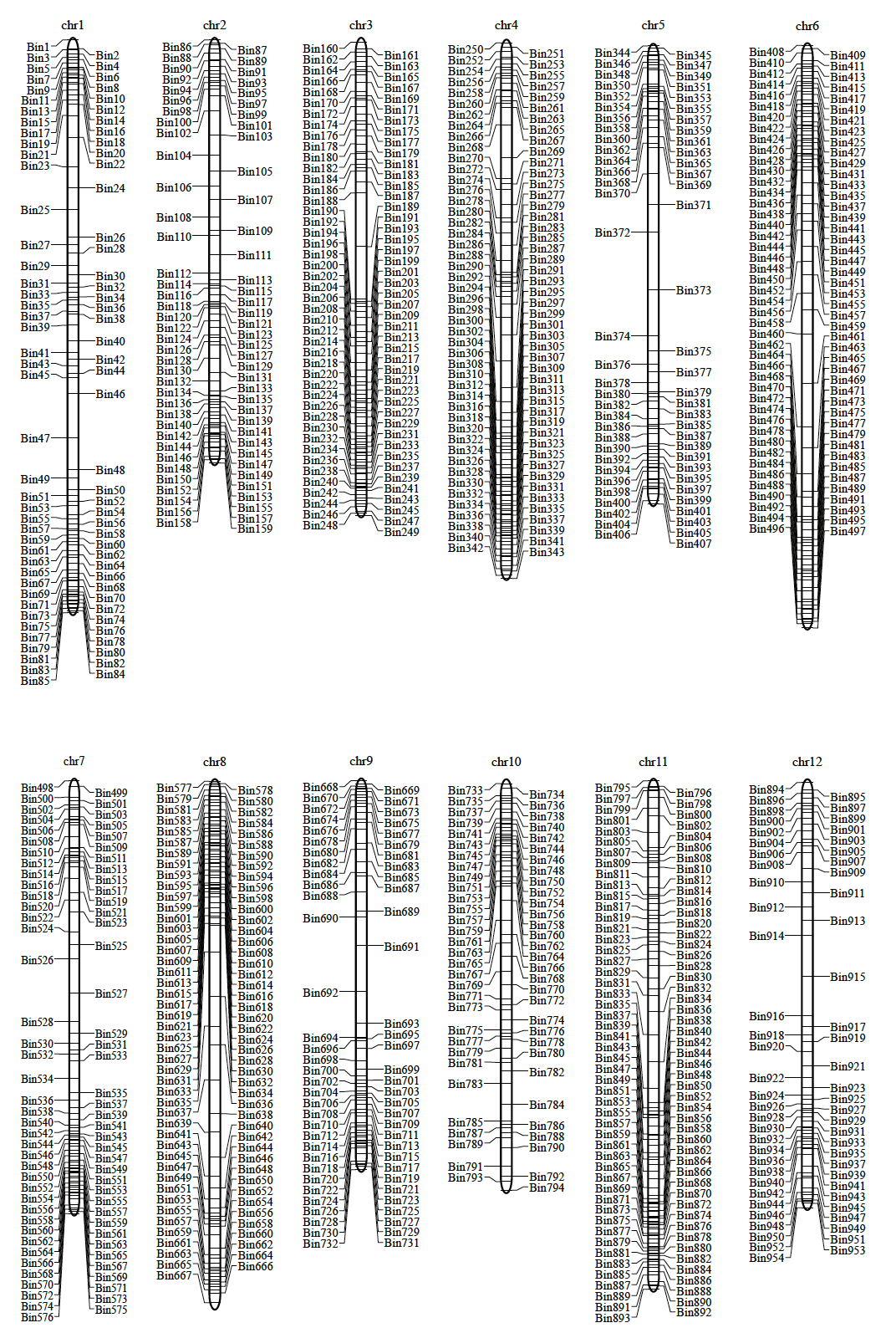


**Fig. S3** The distribution of bins in WAP (‘JL475’ × ‘YS474’).


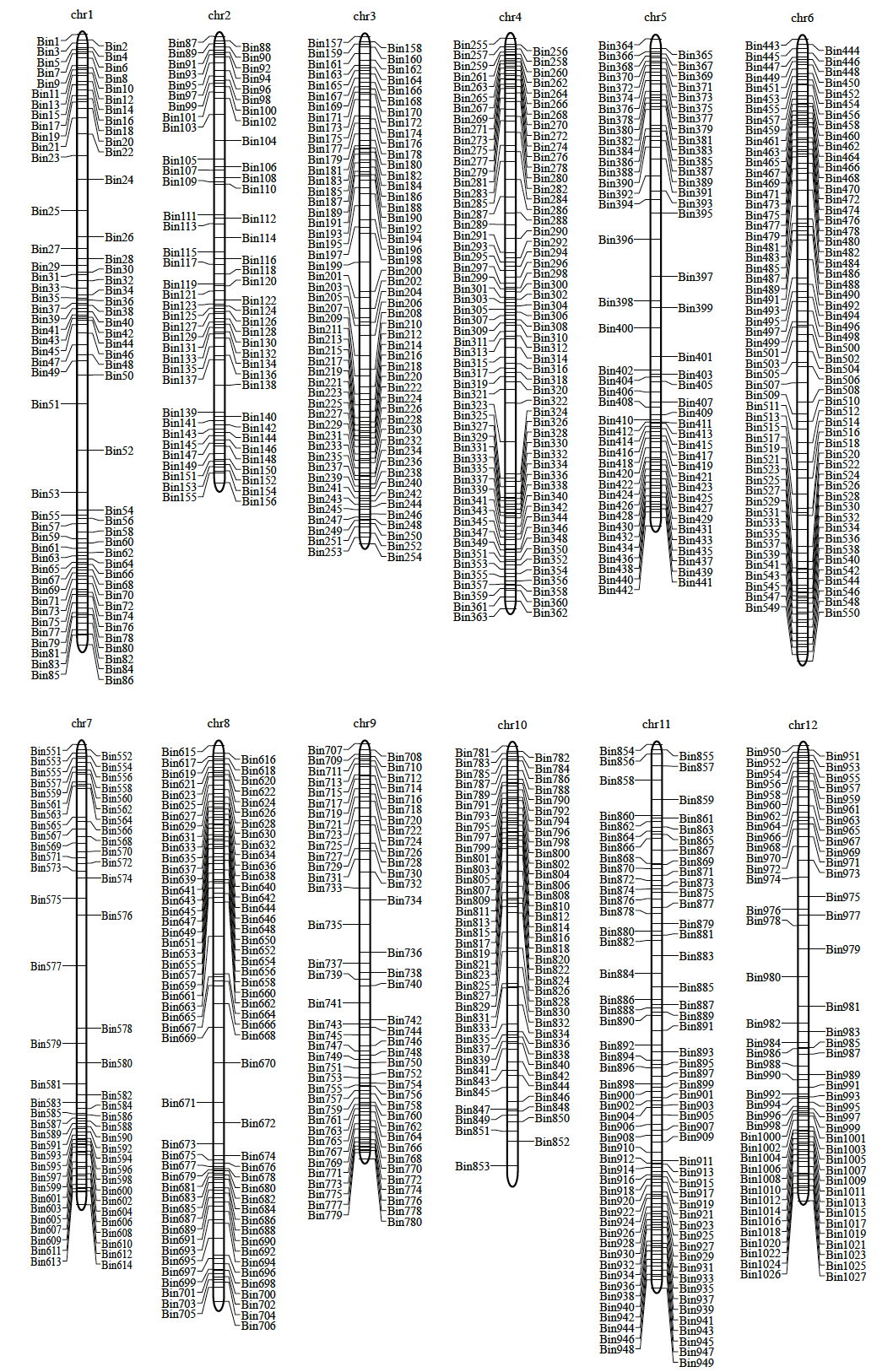


**Fig. S4** The distribution of bins in MAP (‘HG118’ × ‘SD119’).


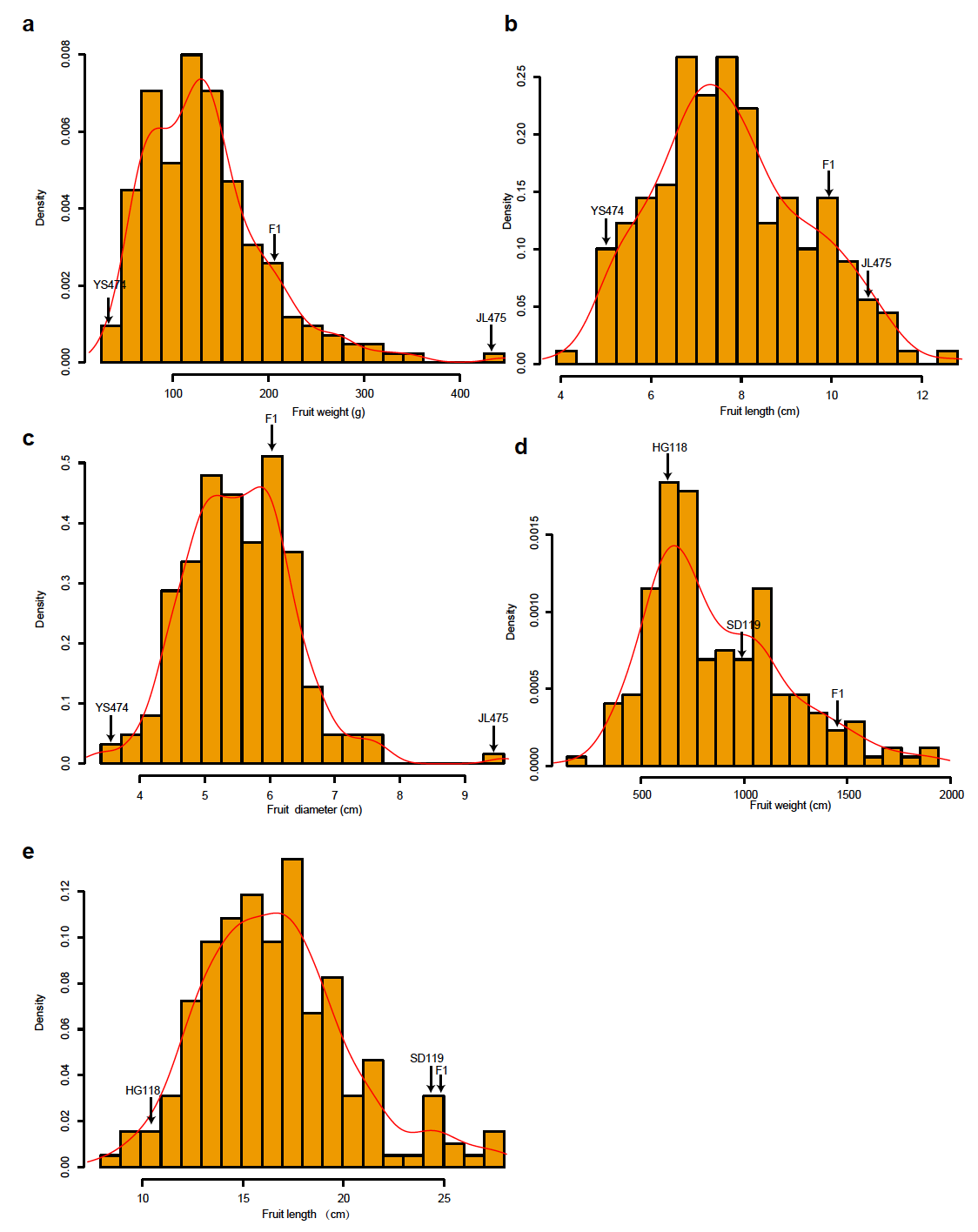


**Fig. S5 Frequency distributions of fruit weight, fruit length, and fruit diameter in two F_2_ populations.**

The distributions of phenotypic values (fruit weight, fruit length, and fruit diameter) in WAP (‘JL475’ × ‘YS474’) (A, B, C) and MAP (‘HG118’ × ‘SD119’) (D, E). The phenotypic values of parents and F_1_ plants were marked by black arrow.


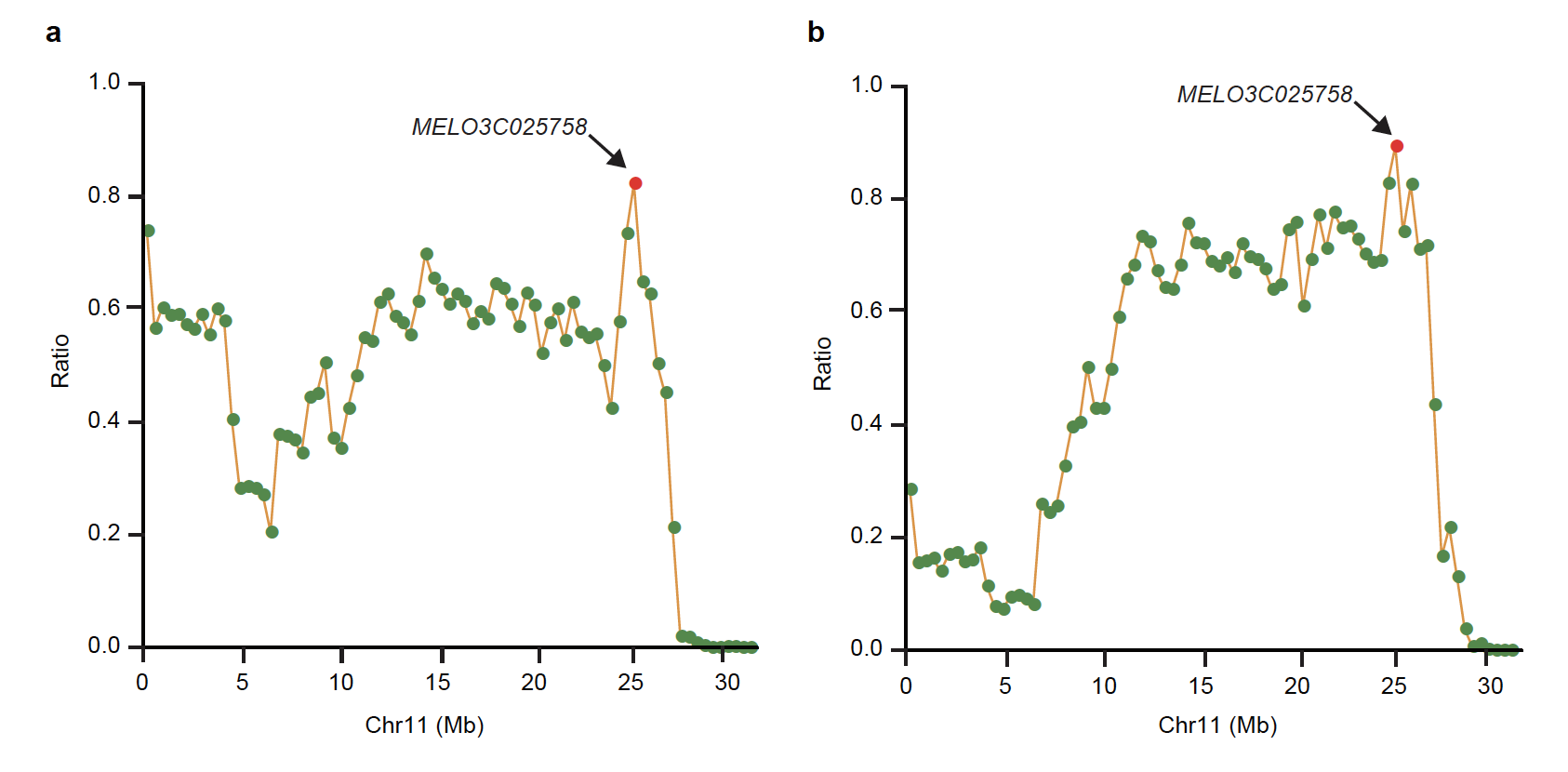


**Fig. S6** GPS-mapping of *MELO3C025758* in WAP.

The genes for fruit weight **(A)** and fruit diameter **(B)** in WAP (‘JL475’ × ‘YS474’). The X-axes values is set at the midpoint of each defined genomic interval, and the Y-axes value corresponds to the ratio. The arrow indicates the location of the peak.

**
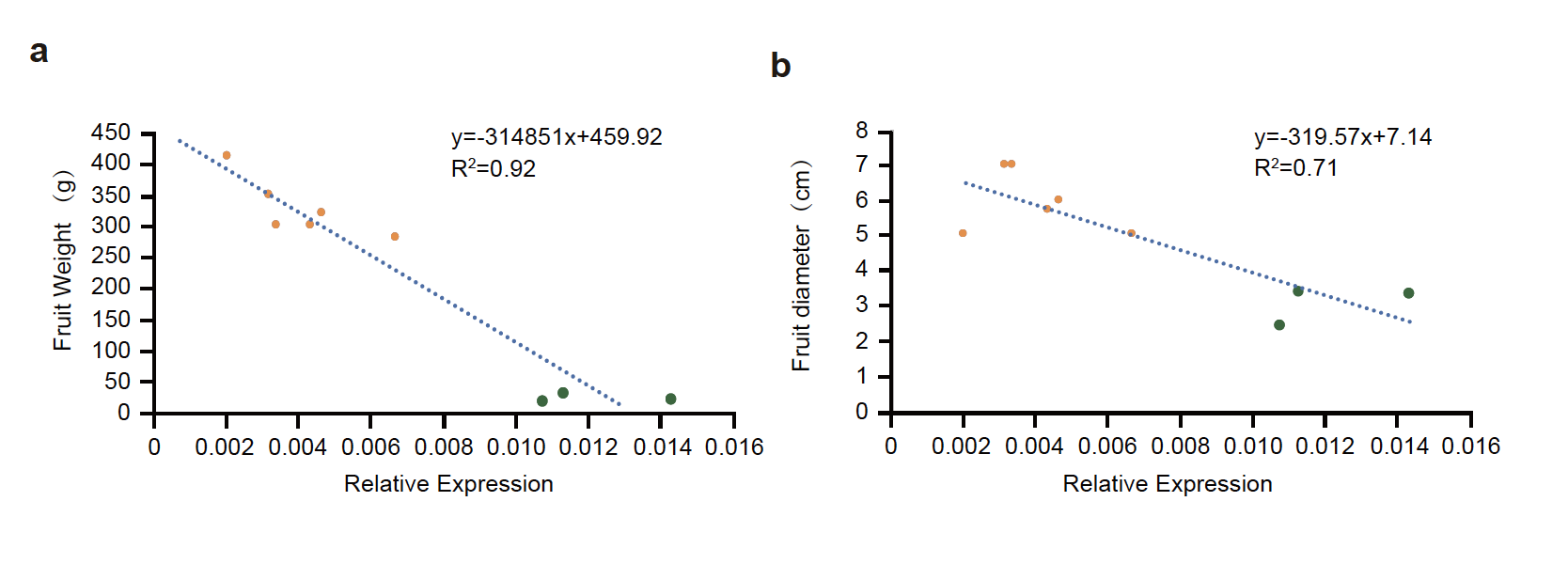
**

**Fig. S7** The correlation between gene expression (*MELO3C025758*) and fruit weight **(A)** and fruit diameter **(B)** in 9 diverse melon accessions including 6 cultivated *agrestis* (Chinese landraces from the group *C*. *melo* ssp. *agrestis* var. *chinensis*) (orange dot) and 3 wild *agrestis* accessions (green dot).

**Table S1** The summary of sequencing statistics

| **Group** | **Name** | **Clean Reads** | **Clean Bases** | **Reads Length (bp)** | **Mean Depth**  **(fold)** |
| --- | --- | --- | --- | --- | --- |
| WAP | JL475 (female) | 78,959,760 | 11,843,964,000 | 150 | 29.11 |
|  | YS474 (male) | 83,051,926 | 12,457,788,900 | 150 | 30.61 |
|  | F_2_ (average) | 18,080,178 | 2,712,026,767 | 150 | 6.66 |
| MAP | HG118 (female) | 79,707,952 | 11,937,372,318 | 150 | 29.34 |
|  | SD119 (male) | 78,098,424 | 11,695,761,728 | 150 | 28.74 |
|  | F_2_ (average) | 20,024,320 | 2,998,621,451 | 150 | 7.37 |

| **Segregation Distortion Region** | **BinID** | **ChID** | **Start** | **End** | **Female Genotype** | **Heterozygote Genotype** | **Male Genotype** | **P-value** |
| --- | --- | --- | --- | --- | --- | --- | --- | --- |
| WASDR1 | Bin106 | chr2 | 8,742,474 | 9,538,595 | 53 | 76 | 67 | 0.00263525 |
| WASDR1 | Bin107 | chr2 | 9,538,596 | 10,261,947 | 54 | 78 | 67 | 0.00410736 |
| WASDR2 | Bin510 | chr7 | 2,993,931 | 4,113,240 | 55 | 74 | 67 | 0.00134381 |
| WASDR3 | Bin762 | chr10 | 6,325,363 | 6,411,889 | 52 | 80 | 67 | 0.00706741 |
| WASDR4 | Bin777 | chr10 | 15,637,626 | 15,787,393 | 51 | 81 | 67 | 0.00886069 |
| WASDR4 | Bin778 | chr10 | 15,787,394 | 16,167,889 | 51 | 80 | 68 | 0.00512375 |
| WASDR5 | Bin780 | chr10 | 16,782,086 | 17,324,084 | 50 | 81 | 68 | 0.00629601 |
| WASDR6 | Bin784 | chr10 | 18,912,869 | 20,906,858 | 52 | 81 | 67 | 0.00878245 |
| WASDR6 | Bin785 | chr10 | 20,911,002 | 21,046,091 | 52 | 80 | 68 | 0.00509243 |
| WASDR6 | Bin786 | chr10 | 21,066,174 | 21,231,369 | 51 | 80 | 68 | 0.00512375 |
| WASDR6 | Bin787 | chr10 | 21,236,567 | 21,483,461 | 51 | 81 | 68 | 0.00637737 |
| WASDR6 | Bin788 | chr10 | 21,488,426 | 21,835,089 | 52 | 81 | 67 | 0.00878245 |
| WASDR7 | Bin790 | chr10 | 22,050,188 | 23,156,909 | 52 | 81 | 67 | 0.00878245 |
| WASDR7 | Bin791 | chr10 | 23,240,787 | 24,201,020 | 49 | 83 | 68 | 0.00914087 |

**Table S2** The SDRs in WAP (‘JL475’ × ‘YS474’)

**Table S3** The SDRs in MAP (‘HG118’ × ‘SD119’)

| **Segregation Distortion Region** | **BinID** | **ChID** | **Start** | **End** | **Female genotype** | **Heterozygote genotype** | **Male genotype** | **P-value** |
| --- | --- | --- | --- | --- | --- | --- | --- | --- |
| MASDR1 | Bin110 | chr2 | 8,655,182 | 8,711,921 | 13 | 135 | 32 | 2.27705E-11 |
| MASDR1 | Bin111 | chr2 | 10,393,817 | 10,481,710 | 16 | 114 | 36 | 8.41608E-07 |
| MASDR2 | Bin116 | chr2 | 12,717,677 | 13,157,407 | 46 | 114 | 28 | 0.002532057 |
| MASDR2 | Bin117 | chr2 | 13,163,112 | 13,326,447 | 47 | 115 | 28 | 0.002219382 |
| MASDR3 | Bin133 | chr2 | 17,157,859 | 17,235,530 | 53 | 114 | 31 | 0.008940429 |
| MASDR3 | Bin134 | chr2 | 17,310,847 | 17,563,489 | 53 | 119 | 27 | 0.000732833 |
| MASDR3 | Bin135 | chr2 | 17,584,000 | 18,252,787 | 52 | 117 | 27 | 0.001035937 |
| MASDR3 | Bin136 | chr2 | 18,281,154 | 18,598,204 | 51 | 117 | 30 | 0.004086771 |
| MASDR3 | Bin137 | chr2 | 18,598,205 | 18,730,359 | 52 | 117 | 30 | 0.004045904 |
| MASDR3 | Bin138 | chr2 | 18,730,360 | 21,542,038 | 53 | 115 | 30 | 0.005207915 |
| MASDR4 | Bin140 | chr2 | 21,760,936 | 22,048,518 | 53 | 116 | 30 | 0.004541616 |
| MASDR4 | Bin141 | chr2 | 22,048,519 | 22,232,033 | 52 | 117 | 30 | 0.004045904 |
| MASDR4 | Bin142 | chr2 | 22,269,396 | 22,519,137 | 50 | 118 | 30 | 0.003459377 |
| MASDR4 | Bin143 | chr2 | 22,604,171 | 22,682,922 | 51 | 114 | 31 | 0.009532376 |
| MASDR5 | Bin146 | chr2 | 23,165,369 | 23,292,946 | 53 | 115 | 30 | 0.005207915 |
| MASDR5 | Bin147 | chr2 | 23,341,281 | 23,480,162 | 51 | 114 | 31 | 0.009532376 |
| MASDR5 | Bin148 | chr2 | 23,487,579 | 23,708,725 | 49 | 112 | 30 | 0.008731329 |
| MASDR5 | Bin149 | chr2 | 23,948,598 | 24,166,390 | 46 | 114 | 31 | 0.008550375 |
| MASDR6 | Bin151 | chr2 | 24,419,896 | 24,453,699 | 53 | 114 | 30 | 0.005949992 |
| MASDR7 | Bin182 | chr3 | 6,506,635 | 6,539,116 | 54 | 68 | 76 | 5.28037E-06 |
| MASDR7 | Bin183 | chr3 | 6,579,040 | 6,614,848 | 54 | 61 | 83 | 6.61892E-09 |
| MASDR7 | Bin184 | chr3 | 6,660,880 | 6,838,755 | 53 | 60 | 86 | 6.50509E-10 |
| MASDR7 | Bin185 | chr3 | 6,859,555 | 6,926,180 | 52 | 61 | 80 | 3.66533E-08 |
| MASDR8 | Bin398 | chr5 | 14,988,859 | 15,429,212 | 57 | 75 | 68 | 0.001054172 |
| MASDR9 | Bin620 | chr8 | 1,270,284 | 1,438,526 | 47 | 116 | 30 | 0.004348937 |
| MASDR9 | Bin621 | chr8 | 1,438,527 | 1,590,703 | 47 | 116 | 29 | 0.002867925 |
| MASDR10 | Bin623 | chr8 | 1,750,101 | 1,885,157 | 46 | 120 | 30 | 0.001940329 |
| MASDR10 | Bin624 | chr8 | 1,902,663 | 1,984,530 | 45 | 123 | 29 | 0.0006153 |
| MASDR10 | Bin625 | chr8 | 2,014,071 | 2,161,955 | 44 | 125 | 29 | 0.000347535 |
| MASDR10 | Bin626 | chr8 | 2,174,288 | 2,301,601 | 43 | 125 | 28 | 0.000186564 |
| MASDR10 | Bin627 | chr8 | 2,450,128 | 2,635,275 | 42 | 126 | 29 | 0.000196365 |
| MASDR10 | Bin628 | chr8 | 2,762,388 | 2,844,306 | 44 | 121 | 34 | 0.005809614 |
| MASDR11 | Bin654 | chr8 | 7,349,280 | 7,396,537 | 60 | 75 | 63 | 0.002840894 |
| MASDR11 | Bin655 | chr8 | 7,411,381 | 7,730,499 | 59 | 72 | 68 | 0.000332944 |
| MASDR12 | Bin710 | chr9 | 875,950 | 986,718 | 43 | 116 | 33 | 0.009209682 |
| MASDR13 | Bin835 | chr10 | 16,016,915 | 16,209,688 | 19 | 111 | 46 | 3.89431E-05 |
| MASDR14 | Bin837 | chr10 | 16,670,157 | 16,720,514 | 25 | 95 | 49 | 0.008977644 |
| MASDR14 | Bin838 | chr10 | 16,760,215 | 16,895,870 | 19 | 105 | 45 | 0.00012674 |
| MASDR15 | Bin919 | chr11 | 25,112,294 | 25,147,929 | 50 | 73 | 74 | 7.29759E-05 |
| MASDR15 | Bin920 | chr11 | 25,205,664 | 25,440,096 | 49 | 67 | 78 | 1.22398E-06 |
| MASDR16 | Bin964 | chr12 | 2,876,001 | 3,418,516 | 51 | 147 | 0 | 1.54075E-16 |

**Table S4** The result of GO enriches of genes in SDRs in WAP (‘JL475’ × ‘YS474’)

| **Category** | **GO ID** | **Description** | **P-value** | **FDR** |
| --- | --- | --- | --- | --- |
| Molecular Function | GO:0008759 | UDP-3-O-[3-hydroxymyristoyl] N-acetylglucosamine  deacetylase activity | 5.93E-05 | 0.00492226 |
| Molecular Function | GO:0033925 | mannosyl-glycoprotein endo-beta-N-  acetylglucosaminidase activity | 5.93E-05 | 0.00492226 |
| Molecular Function | GO:0019213 | deacetylase activity | 0.00087175 | 0.04823658 |
| Biological Process | GO:0009607 | response to biotic stimulus | 1.29E-16 | 3.53E-14 |
| Biological Process | GO:0006952 | defense response | 1.17E-09 | 1.59E-07 |
| Biological Process | GO:0006950 | response to stress | 2.99E-06 | 0.00027248 |
| Biological Process | GO:0050896 | response to stimulus | 0.00015305 | 0.01044554 |
| Biological Process | GO:0009245 | lipid A biosynthetic process | 0.00051298 | 0.01750543 |
| Biological Process | GO:0046493 | lipid A metabolic process | 0.00051298 | 0.01750543 |
| Biological Process | GO:1901269 | lipooligosaccharide metabolic process | 0.00051298 | 0.01750543 |
| Biological Process | GO:1901271 | lipooligosaccharide biosynthetic process | 0.00051298 | 0.01750543 |

**Table S5** The result of GO enriches of genes in SDRs in MAP (‘HG118’ × ‘SD119’)

| **Category** | **GO ID** | **Description** | **P-value** | **FDR** |
| --- | --- | --- | --- | --- |
| Cellular Component | GO:0048046 | apoplast | 6.14E-05 | 0.007 |
| Cellular Component | GO:0005576 | extracellular region | 0.0026513 | 0.1220388 |
| Cellular Component | GO:0005618 | cell wall | 0.00598 | 0.1220388 |
| Cellular Component | GO:0005615 | extracellular space | 0.0062304 | 0.1220388 |
| Cellular Component | GO:0044421 | extracellular region part | 0.0062304 | 0.1220388 |
| Cellular Component | GO:0030312 | external encapsulating structure | 0.0064231 | 0.1220388 |
| Molecular Function | GO:0016762 | xyloglucosyl transferase activity | 9.28E-07 | 0.0002736 |
| Molecular Function | GO:0005215 | transporter activity | 0.0018469 | 0.2724228 |
| Molecular Function | GO:0022891 | substrate-specific transmembrane transporter activity | 0.0070525 | 0.3526331 |
| Biological Process | GO:0006073 | cellular glucan metabolic process | 0.0007943 | 0.1550522 |
| Biological Process | GO:0044042 | glucan metabolic process | 0.0007943 | 0.1550522 |
| Biological Process | GO:0044264 | cellular polysaccharide metabolic process | 0.0011541 | 0.1550522 |
| Biological Process | GO:0044262 | cellular carbohydrate metabolic process | 0.0017921 | 0.1550522 |
| Biological Process | GO:0051179 | localization | 0.0018651 | 0.1550522 |
| Biological Process | GO:0005976 | polysaccharide metabolic process | 0.0022666 | 0.1550522 |
| Biological Process | GO:0006810 | transport | 0.0025478 | 0.1550522 |
| Biological Process | GO:0051234 | establishment of localization | 0.0027262 | 0.1550522 |
| Biological Process | GO:0006644 | phospholipid metabolic process | 0.0053547 | 0.2562556 |
| Biological Process | GO:0006650 | glycerophospholipid metabolic process | 0.005632 | 0.2562556 |
| Biological Process | GO:0046486 | glycerolipid metabolic process | 0.0088053 | 0.3642187 |
| Biological Process | GO:0046488 | phosphatidylinositol metabolic process | 0.0098326 | 0.3728212 |
